# Contesting an exclusive citizenship regime: the Kurdistan Workers Party (PKK) and its electoral mobilisation in Batman in the late 1970s

**DOI:** 10.1080/01436597.2025.2518501

**Published:** 2025-07-01

**Authors:** Joost Jongerden, Francis O’Connor

**Affiliations:** Rural Sociology, Wageningen University, Wageningen, The Netherlands

**Keywords:** Kurdistan, citizenship, activism, PKK, municipal politics, Turkey

## Abstract

Repeated calls have been made to focus on the *politics* of political violence for a better understanding of the political fundamentals of an armed movement. In this article we look into the politics of the Kurdistan Workers Party (*Partiya Karkerên Kurdistan*, PKK) through its engagement in municipal politics in the late 1970s in Batman, then a rural town in the Kurdistan region in Turkey, which rapidly industrialised after the local discovery of oil. Using a citizenship analytical lens, this article makes two substantial contributions. The article challenges overly simplistic, linear narratives regarding the PKK’s origins and its eventual embrace of violence. By analysing the PKK’s electoral and representational politics in the late 1970s, it emphasises the political dynamics of that period rather than reinterpreting its emergence solely through the later insurgency. Empirically, the article illustrates how the Kurdish political movement’s pursuit of representation directly challenged the ethnically exclusionary citizenship regime of the Turkish state.

## Introduction

There have been recent calls to focus on the *politics* of political violence (Staniland 2023), rooted in the premise that the process of decentring violence could lead to ‘different questions being asked, and new unexpected answers being developed’ (Worrall and Waterman 2023, 622). And what better time to concentrate on the political fundamentals of an armed movement than in its formative phase, when political pathways are not yet fully defined (O’Connor and Oikonomakis 2015, 380)? Although, the Kurdistan Workers Party (*Partiya Karkerên Kurdistan*, PKK) has long been a subject of academic interest because of its military capabilities and its stubborn resilience over decades of armed struggle, less attention is afforded to the politics of its early formation. Through an analysis of the PKK’s early activism in the 1970s, this article focuses explicitly on the political imaginary that the PKK constructed in the course of its engagement in municipal politics. Using an ‘activist citizenship’ analytical lens (Isin 2009), the article seeks to outline how the political project advanced by the PKK challenged Turkey’s restrictive citizenship regime. This issue has recently returned to public attention, following the PKK’s decision to dissolve itself and completely end its armed struggle, contingent on Turkey’s recognition of the right to democratic politics – a right whose denial had originally led to the PKK’s formation and eventually a prolonged armed conflict.

By foregrounding the concept of citizenship and the exclusionary nature of Turkey’s citizenship regime, we interrogate the meaning of radical political mobilisation in Turkey. Citizenship regimes are institutionalised practices that define the norms and conditions of and for citizenship (Isin 2024; Jenson 2007). Citizenship confers responsibilities and rights (Vink and Bauböck 2013, 622), as well as delineating the boundaries of political inclusion and exclusion (Isin 2024; Jenson 2007). Though all citizens in Turkey are ostensibly equal before the law, in practice Kurds have historically been able to exercise their rights only as ‘prospective Turks’ (Yeğen 2009, 597). Accordingly, it is through challenges to the state’s citizenship regime that its exclusionary nature is most clearly revealed. We define acts of citizenship as those that challenge restrictive citizenship regimes by making claims to rights (Isin 2024), such as the PKK’s engagement in municipal politics in the late 1970s. The political, then, emerges when those who have been excluded, rendered invisible, or silenced assert themselves and become seen and heard through such acts of claim-making.

Our examination of the PKK’s engagement in political contention through institutional channels like municipal elections makes two distinct contributions. Theoretically, the article argues against an overly simplistic or linear understanding of the PKK’s origins and eventual adoption of violence. By examining the PKK’s actual engagement in representational politics in the late 1970s, it emphasises the political dynamics at the time, rather than retrospectively considering the PKK’s emergence through the lens of the insurgency which later ensued. This does not make the article an exercise in counterfactual reasoning regarding potentially different outcomes or trajectories, if the PKK’s experiences in municipal and electoral politics had not been violently crushed by the state. Yet it does show how escalation to violent conflict does not have a linear or irreversible logic. Empirically, this demonstrates how the Kurdish political movement’s claim to representation challenged the ethnically exclusionary nature of the Turkish state’s citizenship regime. We argue that the PKK – and Kurdish political mobilisation more broadly – has not struggled merely against the state itself, but more specifically against its exclusionary conception of citizenship.

The article begins by giving an overview of general Kurdish electoral mobilisation in Turkey in the 1970s, before proceeding to looking at the nature of state–contender interactions, showing how the PKK engaged with institutional politics in the 1977–79 period. Although the official ideology of the state denied the existence of Kurds, hence, de facto excluding them from electoral and representative politics, the PKK challenged this restrictive citizenship’s regime on its own institutional terrain, by participating in elections. The foregrounding of this institutional politics allows a novel reading – a vital *inversion*, in fact – of the dominant understanding of the struggle of Kurdish political actors in Turkey, which starts from the assumption of political separatism. The study concludes by detailing how this one localised instance of democratic contestation foundered on the shores of state violence and makes some suggestions regarding elements of continuity between the PKK’s mobilisation of this period and the strategies of democratic contestation advanced by Kurdish actors implemented over the last 35 years.

## Theorising citizenship regimes and resistance: framework and methodology

Turkey’s ‘Kurdish issue’ is intimately intertwined with the nature of citizen–state relations in the construction of the nation-state. While many studies on the Kurdish issue in Turkey have focused on the dynamics between the Turkish state and Kurdish armed resistance (Aydin and Emrence 2015, Gunes 2012, O’Connor 2021, Romano 2006), Kurdish participation in electoral politics has received significantly less attention. This is particularly the case for the PKK’s electoral engagement. The few studies that do address this area often focus either on the relationship between representative political actors and the armed politics of the PKK (Gunes 2012), or on the opportunities for new forms of legitimacy, contestation, and resource mobilisation created by representational and electoral politics (Watts 2010). As a result, critical phases of the history of the PKK’s involvement in electoral and representative politics have largely been overlooked. Yet understanding this neglected past is crucial for comprehending how a struggle for recognition and rights eventually escalated into an insurgency. Revisiting this brief period of Kurdish electoral engagement helps us recognise alternative political pathways that were not pursued before the 12 September 1980 military coup and the subsequent onset of the PKK’s armed struggle.

The late 1970s were marked not only by the concerted engagement of various strands of the radical left with politics in Turkey (Houston 2020; Lipovsky 1992), but also by the involvement of Kurdish political movements in institutional politics. During this period, several Kurdish and leftist political organisations supported candidates who went on to win municipal elections in numerous cities and towns. The PKK, for instance, successfully ran mayoral candidates in the 1979 municipal elections in Batman and Hilvan, with Edip Solmaz and Nadir Temel respectively elected (O’Connor and Jongerden 2023). In Diyarbakır, the Socialist Party of Turkish Kurdistan (*Türkiye Kürdistan Sosyalist Partisi*, TKSP) supported Mehdi Zana, who won the mayoral election in 1977 (Zana 1997). Another TKSP-backed candidate, Urfan Alparslan, secured a victory in the 1979 by-election in Ağrı (Yörük 2016). In a notable non-Kurdish case, Fikri Sönmez was elected mayor of the Black Sea district of Fatsa with the support of Revolutionary Way (*Devrimci Yol*, DEV-YOL), a radical leftist organisation aiming to transform Turkey’s class structure (Acaroglu 2019). However, none of these mayors served their full terms in office. Edip Solmaz was assassinated, while Nadir Temel, Orhan (Urfan) Alparslan, and Fikri Sönmez were removed from office by the Ministry of Internal Affairs, imprisoned, and replaced by state-appointed trustees. The removal of elected Kurdish mayors and their replacement with unelected trustees has become a recurring state practice, particularly widespread since 2016 (Hintz and Ercan 2024; Tutkal 2022; Whiting and Kaya 2021).

This securitisation of institutional politics inevitably raises the question of citizenship – not only who is able to exercise rights and who is not, but also how citizenship itself operates as an apparatus of governance, a mechanism through which power is exercised and insulated from those who make claims against it. Citizenship, through institutions, policies and practices, regulates the conditions under which individuals are recognised as citizens and mediates their relationship with the state (Isin 2024). As a governance tool, citizenship not only defines rights and duties but also establishes who is allowed to belong and who is excluded. In his historical analysis of citizenship practices in Turkey, Mesut Yeğen (2009) introduces the concept of the ‘prospective Turk’ to describe the citizenship status of Kurds in Turkey. His concept highlights the ambiguity of Turkish citizenship, which is not only a legal category but in practice also an ethnic one. Yeğen (2004) argues that Kurds have been positioned as ‘outsiders’ to the Turkish nation to whom citizenship rights do not fully apply. Instead, they could exercise their citizenship only as ‘prospective Turks’ (Yeğen 2009). In other words, political participation by Kurds is contingent upon their cultural self-qualification as ‘Turks’ (Barkey 1998) and their alignment with the state (Bozarslan 2008, 2019).

As a result, Kurds have been subject to both assimilation policies that were designed to turn them into Turks, and security-driven governance, which have curtailed their rights. As an assimilative practice, citizenship obtained a ‘civilizing mission’ (Mamdani 2020, 61) or what is referred to as a process of ‘internal colonization’ (Jongerden 2007, 198) that had to convert Kurds into ethnic Turks (Bruinessen 2016). However, the prospective nature of ‘prospective Turkishness’ leaves open the possibility that Kurds may not fully assimilate into a Turkish national identity. This is evident in the exceptional political governance of Turkey’s Kurdish south-east, which has frequently been subjected to emergency rule, states of exception, or trusteeship since the founding of the Republic in 1923.

Through their engagement with institutional politics, Kurdish political movements have challenged the very terms of citizenship, pushing against the boundaries imposed by the state and its governance apparatus. By fielding candidates in municipal elections in the late 1970s, the PKK and several of its Kurdish contemporaries (O’Connor and Jongerden 2023) posed an unprecedented challenge to Turkey’s existing ethno-nationalist citizenship regime. This political intervention coincided with a series of structural developments that had been reshaping Turkey’s political landscape: political instability at the national level, marked by repeated inconclusive elections (Gunter 1989), intensifying inter-elite competition (Somer 2016), fierce struggles between landlords and villagers around the issue of land reform (Gürel, Küçük, and Taş 2022), and the emergence of working-class struggles driven by industrialisation (Keyder 1987). This proved to be a fertile ground for radical political ideologies that aimed to reshape the political system in Turkey (Akkaya 2013; Jongerden and Akkaya 2019). However, due to legal restrictions prohibiting candidates from campaigning on explicitly pro-Kurdish platforms, Kurdish parties and movements – operating in a semi-clandestine fashion – had to formally field candidates as independents. On the ground, however, there was no ambiguity about these candidates’ affiliations. We consider this form of participation in electoral politics an act of citizenship (Isin and Nielsen 2008), through which the PKK interrupted an existing citizenship regime and established Kurds as bearers of rights.

### Data and analysis

In general, the first years of the PKK’s political formation and mobilisation are characterised by data scarcity and accessibility (O’Connor and Akin 2023). This is even more pronounced for the earliest years of the PKK’s emergence prior to the 1980 coup. There are very limited written primary sources on the daily activities and political practices of the movement in this period. The few available sources are in the form of memoirs and commemorative documents (Ekinci 2013), articles published in the PKK’s newspaper Serxwebûn (1982, 1983) or other outlets close to the Kurdish movement (Eren 2019; Yaş 2019).

On the basis of a case study in Batman, this article presents unique qualitative data about the politics of the PKK in its early years. This data was gathered through a series of interviews with (former) PKK activists, militants from other parties and active movements, and first-hand observers and witnesses of the period. It contextualises this original data in the available movement documentation and media coverage of the period, as well as relevant institutional archival materials and census data. A total of 23 interviews (19 men, four women) were conducted by one of the co-authors between February 2022 and December 2023 in various locations in Europe and Turkey (see Online Appendix A1). Most of the interviewees were in their 60s and 70s at the time of interview: 20 had been either activists in the PKK or politically involved in other local political elite groups, rival political factions and movements. As Batman was still a relatively small town in the 1970s, it is theoretically possible that informed parties could potentially identify interviewees; accordingly, the authors adopted a strict interview anonymisation protocol, limiting the level of detail that has been made publicly available. Three additional interviews were conducted with experts with specific political and economic knowledge of the 1970s in order to facilitate the triangulation of findings. The interview data build on the accumulated knowledge that the authors have acquired through previously conducted periods of fieldwork, which have addressed associated topics in the same time period. In addition to its innovative application of a new theoretical perspective to the subject, it makes a unique empirical contribution to the understanding of the PKK during this period, advancing and presenting a more nuanced understanding of the movement’s repertoire of contention in its earliest years.

Although Kurdish movements participated in several elections across the region in the late 1970s, the Batman election is the focus of this article for two main reasons. Firstly, the PKK’s campaign in Batman reveals much about broader political patterns in Turkey where industrialisation, intra-elite competition, migration, peasants’ and workers’ struggle, and political radicalisation created the ground for a range of political movements to challenge existing models of political governance. Secondly, it challenges the linear interpretation of insurgent trajectories, emphasising how such readings often overlook the importance of interactions and contingency during the early stages of mobilisation (see Malthaner and Malešević 2022; O’Connor and Jongerden 2023).

## Batman: intra-elite political struggle

Situated between the cities of Diyarbakir and Siirt, Batman (Iluh in Kurmancî) was originally a small village of only 319 residents in 1935 (Sunkar and Tonbul 2011). By the 1970s, the population had increased to 55,000, and by 1980, it had further grown to 86,000, with approximately 80% of the district’s residents living in the city (see Table 1). The fundamental reason for this rapid growth was the booming oil industry since the 1950s^1^ and the construction of a railway connecting Batman with labour reserves beyond the surrounding villages and towns (Oguz 2021; Yildiz 2019).

**Table 1.** Population of Batman, 1970–80 (Turkish Statistical Institute 1970 and 1980 General Population Census Database).

	1970	1975	1980
City	44,991	64,384	86,172
Villages	12,276	14,209	16,631
Total	57,267	78,593	102,803

The Raman, a large tribe in the southern parts of Batman, were economically boosted by the extraction of oil on their lands. Their growing economic power eventually extended into governance when Batman was officially established as a separate municipality in 1955. The Raman assumed political and administrative authority, taking control of the city’s municipal resources and gaining the power to regulate land use and development through the issuing of construction permits – key instruments for accumulating wealth and power. The Ramans’ accelerated strengthening upset the prevailing local balance of power, particularly disquieting its rivals, the Zilan, a tribe dispersed over a territory to the north of Batman. Troubled by what they referred to as the Raman ‘sultanate’ (Interview #8), the Sheikh (*Şeyh*) of the Zilan sought political backing to counter the burgeoning influence of the Raman, and found support from the Republican People’s Party (*Cumhurriyet Halk Partisi*, CHP), the national competitor of the Justice Party (*Adalet Partisi*, AP), with which the Raman were aligned at the time.

In the 1970s, politics in Kurdistan was overwhelmingly dominated by traditional tribal elites who offered support for national political parties in return for access to resources to consolidate their local political and economic interests (Barkey and Fuller 1998, 77; Klein 2012) – but, importantly, without challenging the existing citizenship regime. As mentioned, Batman’s rapid urban growth and oil wealth had brought various local elites into conflict with one another. The Zilan, seeking possibilities to counter the expanding political and economic power of the Raman, found an ally in the CHP, which was touring the region to garner support against the Justice Party, the ally of the Raman. According to one of the interviewees, CHP leader Bülent Ecevit met Sheik Zilan who apparently proposed to Ecevit that if the CHP nominated Şahabettin [Bağdu] as their candidate for the municipal elections, his many followers [*mürit*] would vote for the CHP (Interview #8). By aligning themselves with the CHP, the Zilan obtained central state political support to challenge the intertwining of the Raman and state power at the local level. Şahabettin Bağdu was closely related to the Sheikh, but as a state bureaucrat he was also viewed as an acceptable candidate by the secular CHP. Bağdu had embarked on a military career, making the grade of lieutenant until he was stripped of his rank and discharged after participating in a failed coup in 1963 (Interview #6).^2^ After having been forced to leave the army, he studied public administration (*mülkiye*) at the Political Sciences Faculty (*Siyasal Bilgiler Fakültesi*) in Ankara, where he graduated in 1968, before becoming an inspector (*müfettiş*) at the Ministry of Development and Housing (*İmar ve İskan Bakanlığı*).

Sheikh Zilan successfully mobilised his followers to vote for the CHP. One interviewee, who had been involved in Zilan’s election campaign and had helped protect the polling stations – later becoming part of Şahabettin Bağdu’s security detail – recalled:

At that time, we were fighting a great struggle against the Raman tribe. […] From the time Batman was founded until the elections of 1977, Batman was in their hands. We fought the fight […] Şahabettin Bağdu became elected as mayor, and they [the Raman] were in shock. (Interview #6)

As Bağdu and his supporters were to learn, however, winning the election was one thing, but maintaining control over the municipality was quite another. Out of fear of Raman retaliation, Bağdu had to be kept under permanent protection. The tribes were tethering at the brink of armed conflict when Mustafa Öztüzün, a strongman in the Zilan tribe, was ambushed and fatally shot (Interviews #5, #6). An interviewee recalled:

I said, ‘We will take revenge. What needs to be done will be done’. I said, ‘I’m ready, whatever you do’. But the Sheik looked at the situation and saw that the tribes have come to the point of war, so the Sheik decides to withdraw from the confrontation. As a result, there is no one left with Şahabettin [Bağdu]. The whole family let him down. […] I said, ‘We’ll do whatever we can. We’ll try to protect you. If someone tries to shoot you, we’ll prevent it. We’ll do whatever we can’. Şahabettin decided otherwise. ‘If they let me down’, he said, ‘I won’t stay as mayor’. He resigned. (Interview #6)

Bağdu’s resignation triggered the 14 October 1979 by-election, presenting the PKK with a key political opportunity: the chance to perform a significant ‘act of citizenship’ – not merely enacting a ‘political fiction’ (Bocheńska et al. 2025) – but one with very real consequences for local power: running for the position of mayor.

## New municipal actor: the PKK/Kurdistan Revolutionaries

When the state’s power is enacted through different constellations of institutional (party) and local elite (agha, sheikh) networks, an excess of statehood practices may occur, with ‘too many actors competing to perform as state’ (Aretxaga 2003, 396). This was the case in Batman, where dysfunctional alliances between national parties and local elites created an opening for radical Kurdish political mobilisation. As the struggle between the Raman and Zilan was unfolding, a new political actor had entered the scene in Batman: the Kurdistan Revolutionaries (*Kurdistan Devrimcileri*), which subsequently became known as the Kurdistan Workers Party (Partiya Karkerên Kurdistan, PKK) after its formal establishment in late 1978 (Jongerden and Akkaya 2012).^3^ As most of the attention in the 1977 Batman election centred on the tribal struggle, the participation of the PKK’s independent candidate, Ibrahim Ramanlı, passed unobserved – especially because he received only 127 votes and finished in last place. Yet the movement considered it an encouraging first step. Having only recently begun organising in Batman, they viewed each voter as a potential bridgehead into a family and the broader social environment (Interview #20).

From 1977, the PKK had stationed prominent cadres in Batman, among them founding members Haki Karer, his brother Baki Karer and Mazlum Doğan. To boost morale and provide the struggle with momentum, PKK leader Abdullah Öcalan visited Batman at least twice in 1977, further attesting to the importance the movement attributed to the city (Interviews #1, #3). The PKK’s cadres established a solid base that comprised some 15–20 full-time activists,^4^ which provided the organisational core for both its municipal politics campaign and other forms of mobilisation.

In their early years, other political parties regarded the PKK with disdain:

The other organisations looked down on the Kurdish Revolutionaries. They were portrayed as the children of workers and shepherds, the children of poor families. […] But this is a feudal thing, you know, where there is a strong divide between the ‘haves’ and the ‘have-nots’. The ‘haves’ have rights, the ‘have-nots’ don’t. The ‘haves’ have the right to express oneself, the right to be listened to, to be heard, and the ‘have-nots’ don’t. (Interview #9)

However, it didn’t take long for the PKK to successfully challenge the other parties. This was largely due to the political climate that permeated revolutionary politics in the 1970s. Kurdish and leftist organisations found themselves mired in sectarian debates over issues such as whether Russia was revisionist, whether China was opportunistic, what Marx truly said and so on (Interview #3). The PKK was less concerned with these debates and instead focused its energy on understanding the political status of the Kurds, and how to change it. Senior PKK cadres in Batman sought to cultivate a political consciousness that linked the struggle against the state as a political oppressor with the struggle against landlords as an exploitative comprador class. As a key local organiser, Mazlum Doğan^5^ organised house meetings where small numbers of people would meet on a regular basis. These gatherings featured intensive reading and discussion sessions, where the principles of Marxism–Leninism were examined in relation to the national struggle in Kurdistan and the alliance between the state and the comprador landlord class (Interview #3). Crucially, these sessions also emphasised what interviewees (Interview #1, #2, #20) referred to as ‘*terbiye*’ – a Turkish term that translates roughly to ‘manners’ or ‘posture’. It refers to the conduct expected of revolutionaries in their daily lives: a rejection of gambling, theft, prostitution, and addictive substances, along with respectful behaviour when visiting homes. This included attention to bodily comportment, such as how to sit and how to interact appropriately with women in the household, and the party. In short, *terbiye* fostered a political consciousness that was closely linked to strict ethical personal behaviour.

New members were recruited through personal contacts, often followed by house visits – not only within the city, but also in surrounding villages – to engage with family members and neighbours. This grassroots strategy led to an expanding circle of sympathisers and the consolidation of a supportive constituency (O’Connor 2021, 3). The PKK’s growing influence became increasingly visible in various local associations. One notable example was the *Batman Devrimci Liseliler Derneği* (Association of Revolutionary High School Students in Batman). On first sight, a former high school member recalls, the place they rented looked like a typical teahouse (Interview #3), except the posters displayed on the walls – such as one of PKK martyr Haki Karer (Interview #1) – revealed the political leanings of its members. Similarly, the PKK extended its influence into the Teachers Union (TÖB-DER) and the Petrol-Workers’ Union (*Petrol-İş Sendikası*).^6^ It was through these spaces – homes, associations, unions, neighbourhoods, and the city itself – that Kurdistan revolutionaries cultivated routine interactions with both existing and potential supporters (Jongerden 2017; O’Connor 2021). By embedding their activities across these multiple and overlapping spatial dimensions, the PKK succeeded in establishing itself as a dominant local political force – an influence that continues in the city to this day.

## Edip Solmaz: from the military to politics

By 1979, the PKK had built a notable support base, primed to seize the political opportunity that arose in the form of the by-election. Yet, to be able be able to participate in the elections as an ‘act of citizenship’, the PKK had to find a candidate who met the formal procedural criteria: being more than 27 years old and having completed their military service (Interviews #8, #11). As most of the PKK’s activists were in their late teens and early 20s, and had not finished their military service, there was a shortage of suitable candidates within its own ranks, leading the PKK to settle on Edip Solmaz as its candidate.

Solmaz came from a family that was known to support the political struggle of the Kurds. Born in a village close to Batman, he was raised and went to school in Batman, after his father found a job as a worker at the Turkish Petroleum Corporation (*Türkiye Petrolleri Anonim Ortaklığı,* TPAO) in 1955 (Interview #4). Solmaz and his family were thus the very embodiment of the rural–urban migration which shaped Batman as a new political frontier. After attending primary (*Devrim Okulu*) and secondary school in the compound of the oil industry (*Petrolkent Site Okulu*) in Batman, Solmaz attended the Kuleli Military High School (*Kuleli Askerî Lisesi*) in Istanbul, and then the Military Academy (*Kara Harp Okulu*) in Polatlı, Ankara (Interview #4). Following his graduation from the academy, Solmaz was stationed in Diyarbakir, from September 1970 until his discharge in March 1977, holding the rank of first lieutenant (Ekinci 2013).

While in the military, Solmaz already had personal connections with people active in the Kurdistan Revolutionaries. Selahattin Çelik, the PKK’s aforementioned candidate in the Petrol-Workers’ Union elections, and Solmaz knew each other because their families were neighbours. Moreover, Solmaz had not hidden his political sympathies and had visited imprisoned activists while he was still in the military. One activist from that time remembered:

I was in prison twice [in 1976–77]. Once for two months, once for three months. Edip Solmaz came to see me many times and brought me cigarettes. He was an officer. I mean, he was a soldier, an officer, a first lieutenant in the Turkish state. He brings us these packages of armed forces cigarettes. He came to visit us personally and brought us these cigarettes. He was our friend. […]

[Interviewer] Was he in the army then?

Of course, he was an officer. He told us he experienced problems in the army. He told us that backward officers in the army were responsible for maintaining the status quo in Turkey but that there was a countermovement, too [of progressive officers]. He told us the state wanted to expel the progressive officers. (Interview #3)

His candidacy for mayorship symbolised a more general transition to electoral politics as a site for change within radical political movements in Turkey. In the late 1960s and, to a lesser degree, in the 1970s, there had been a strong belief among some socialist groups that reputed allies in the Turkish army would emerge to back revolutionary movements in a strategy referred to as the National Democratic Revolution (*Milli Demokratik Devrim*, MDR).^7^ Prominent thinkers within the revolutionary left in the 1960s and early 1970s, such as Mihri Belli, Muzaffer Erdost and Hikmet Kıvılcımlı, had thought of the progressive military as a natural partner in their revolutionary struggle for change (see Lipovsky 1992; Samim 1981; Ünal 1998). But the experience of the 1971 coup and the subsequent repression of progressive, student and revolutionary movements undermined this belief (O’Connor 2021, 36). Solmaz’s individual political trajectory echoes this shift away from any hope in radical political change supported by the army. According to people close to him, the 1975 earthquake in the Kurdish region of Lice was a tipping point. With a strength of 6.6 on the Richter scale, the devastating earthquake destroyed the town and killed more than 2000 people. Deployed there as a lieutenant, Solmaz took part in search and rescue operations and was exposed to the derogatory and dehumanising language used by his army colleagues, such as references to the Kurdish victims as ‘carcasses’ to be done away with (Interview #9). However, he could not leave the army as the completion of 15 years of compulsory service was a condition for officers graduating from a military academy (Interview #8).

He did not resign – it wasn’t possible to resign from the army – so he deserted. After staying two months in hiding in the homes of friends in Batman, he gave himself up. He was taken to the military court, sentenced to four months in prison, and discharged. After he was released, he returned to civilian life. This was in 1978. (Interview #4)

Solmaz had hidden from the authorities at an uncle’s home in the Bahçelievler neighbourhood of Batman, which had been newly constructed in 1977 to provide affordable housing for workers in the oil industry (Interview #9). The fact that Solmaz had sought refuge in such a new workers’ and rural migrants’ neighbourhood highlights the importance of the socio-spatial dynamics of political contention in rapidly urbanising societies (see Houston 2020). After being released from prison, he moved to Istanbul to study law but returned to Batman in 1979 to stand in the October by-election. After his release from prison his candidacy was proposed for approval to the movement’s local constituency, among these the Petrol-Workers’ Union (*Petrol-İş Sendikası* or *Petrol-İş*), the Teachers Union (TÖB-DER), Revolutionary Association of High School Students in Batman (*Batman Devrimci Liseliler Derneği* or *Liseliler Der*) – even though its members were too young to vote – and in the villages that fell within Batman’s municipal boundaries (Interview #3).

## The 1979 by-election

The PKK had rented an office behind the main mosque (*Ulu Camii*) in Batman and opened an election office (*Seçim Bürosu*) – to organise the election campaign. The opening of an election office for the support of their candidate, in the centre of the city, was a symbolic act in which they established themselves as entitled to the right to participate in elections, a concrete ‘act of citizenship’ (Isin 2009). According to the interviewees, Baki Karer,^8^ who had joined the movement in its formational years in Ankara, played an important role in the campaign. He did much of the coordination work, overseeing the reports from the dozens of committees that had been established in different neighbourhoods and district-villages (Interview #1).^9^

Coffeehouses had been turned into offices. In every neighbourhood, everywhere, our committees were working for Edip Solmaz. I was surprised. I hadn’t seen that kind of work, that kind of enthusiasm before. The committees were working until 11 or 12 o’clock at night. (Interview #3)

Another activist recalled how they turned homes and neighbourhood spaces into political spaces. They became what we might call sites of citizenship – spaces around which political contestation gathers (Isin 2009):

We campaigned among the workers of the oil fields. We visited coffeehouses one by one. We went to the neighbourhoods and villages and visited people at home. One by one, we visited the schools. One by one, we visited the shopkeepers and markets, we visited the mosques. Months of intensive campaigning. (Interview #2)

The fields, coffeehouses and homes became spaces where people asserted themselves as citizens and began to challenge an exclusionary citizenship regime. In his campaign, Solmaz positioned himself as the candidate of the people and a representative of progressives and democrats.

‘At the rallies, he said, ‘I’m not the child of a tribe, I’m not an agha, not a bey [landowner], not a sheik. I’m a child of the people. I am the candidate of democrats, patriots, progressives, revolutionaries’ (Interview #7)

Interviewees refer to his term as ‘people’s municipalism’ (*halkçı belediyecelik*), a new form of subaltern municipal politics voicing their demands towards the state, landlords and industry (Batuman 2014; Houston 2020). As the son of a worker in the oil industry, Solmaz positioned himself as one of the people, a distinctive marker in the era of aghas and sheiks, who considered themselves above, rather than of, the people, as members of an elevated, even divine, societal category that set them apart.

Through such rhetorical claims Solmaz expressed a ‘desire for citizenship’ (Bayat 2013, 84), in which revolutionary rhetoric was intertwined with practical interventions in domains that affected the daily lives of people. While the movement he was part of mobilised around a discourse of anti-feudal and anti-colonial liberation struggle, it also intervened in domains that affected people in their daily lives, thereby engaging in acts that concretely challenged the power of the local elites. The militants wrested control of recruitment in the oil workers’ union from the Raman, enacting fair recruitment policies by implementing a lottery system for prospective workers (Interview #2, #7). Thereafter, the names of the applicants were put in a bag, and lots were drawn to determine who was to be hired, thus taking the recruitment power out of the hands of the Raman (Interview #2). In the surrounding countryside the PKK was able to mitigate some of the landlords’ worst excesses, by prohibiting violations of peasants’ and agricultural workers’ homes and enforcing better payment for agricultural labour (Interview #20). In the city, the movement pushed back against the speculation of staple products such as flour and oil, sometimes by raiding and distributing warehoused stocks, thereby enacting the right to affordable food.

The commitment to practical activism that had predated the PKK’s electoral campaigning in Batman was evident in Solmaz’s first initiatives after his election. With winter approaching, Solmaz immediately tasked the municipality to bulk-buy coal, used to heat homes, aiming to make the inhabitants of Batman less dependent on traders and less vulnerable to price speculation. Interviewees also indicate that following his election, centralised distribution of food stuffs, most notably sugar, had begun to be organised. The scarcity of affordable food resulting from speculation had been a central problem in Batman’s daily life, and the PKK underscored this issue throughout the campaign. Interviewees pointed to Solmaz’s pre-election agenda, which aimed to systematically address market speculation by establishing a municipality-regulated market for essential consumer products (Interview #4). It is evident that the PKK garnered respect through their interventions in markets, aiming to disrupt local elites’ control over labour and speculative commodity prices. Besides its advocacy for fair recruitment policies or equitable food prices, it had also shown its capacity to enforce them, and winning the election would have provided it with additional means to do so. In the context of the power struggles between elites in Batman, which exposed fractures in the prevailing state-elite power structure, it was the PKK’s demonstrable record on the ground that inspired hope that the party could effect meaningful change.

## By-election and aftermath

In the Batman municipal by-election of 14 October 1979, the PKK candidate Edip Solmaz received 3876 votes against 3677 for Fahrettin Özdemir, the Raman representative running, once again, on the AP ticket. These were followed by CHP candidate Şevki Akın, who had temporarily taken up office after Şahabettin Bağdu resigned; the independent candidate Abdulgani Demir, who was supported by the Kurdistan Democratic Party (*Kurdistan Demokrat Partisi*, KDP); the independent candidate Lütfü Bakşi, supported by the Kurdistan National Liberators (*Kürdistan Ulusal Kurtuluşçuları,* KUK); the Nationalist Movement Party (*Milliyetçi Hareket Partisi*, MHP) candidate, Şeymus Demir; and two candidates supported by smaller parties, as shown in Table 2.

**Table 2.** Results of the 14 October 1979 Batman by-election.

Candidate	Party	Votes	Percentage
Edip Solmaz	Independent (PKK)^a^	3876	30
Fahrettin Özdemir	AP^b^	3677	29
Şevki Akın	CHP^c^	2045	16
Abdulgani Demir	Independent (KDP)^d^	1070	8
Şeymus Demir	MHP^e^	894	7
Lütfi Baksi	Independent (KUK)^f^	751	6
(Unidentified)	SDP^g^	353	3
(Unidentified)	CGP^h^	222	2
Total		12,897	100

^a^ PKK: Kurdistan Workers Party (Partiya Karkerên Kurdistan)

^b^ AP: Justice Party (Adalet Partisi)

^c^ CHP: Republicans People’s Party (Cumhurriyet Halk Partisi)

^d^ KDP: Kurdistan Democratic Party (Kurdistan Demokrat Partisi)

^e^ MHP: Nationalist Movement Party (Milliyetçi Hareket Partisi)

^f^ KUK: Kurdistan National Liberators (Kürdistan Ulusal Kurtuluşçuları)

^g^ SDP: Socialist Revolution Party (Sosyalist Devrim Partisi), a 1975 break-away from Workers Party of Turkey (Türkiye İşçi Partisi, TİP).

^h^ CGP: Republican Security Party (Cumhuriyetçi Güven Partisi), a 1967 nationalist and anti-socialist break away from the CHP.

On the day Solmaz won the mayor’s office, a rumour spread that the Raman had declared they would prevent him from receiving his first salary (Interview #1), implying he would be killed within a month. The threats against Solmaz were not taken lightly. Mahsum Korkmaz,^10^ one of the young cadres of the movement in Batman, was assigned to protect him, but as an armed former soldier, Solmaz thought he did not need personal protection. His assistant, Talat Yetkin (Ekinci 2013), recalls that on 12 November 1979, they had left their office together and visited the Post and Telegraph Agency (*Posta ve Telgraf Teşkilatı*, PTT), for an update on a telephone connection they had requested. On their way back home, Yetkin invited Solmaz to have dinner together, as they had an evening appointment with a contractor regarding the construction of a sewage connection. However, Solmaz wanted to go home, because his father-in-law and wife had just returned from a trip to Istanbul. He parked his car and had started walking towards his front door when he was shot five times.

Solmaz was killed by an ‘unknown assailant’, but the belief quickly spread that the state had him killed and it was the Raman who had pulled the trigger (Interview #8). The PKK decided to take revenge and targeted Ahmet Özdemir, head of the Ramans’ private militia:

A week passed. Ahmet Özdemir was killed, together with two of his men. The rumor had spread that he was going to be killed. It was being openly talked about, but despite this, Ahmet Özdemir told everyone who wanted to hear that he wasn’t afraid, that nobody could kill him. ‘Who can kill me?’ he said, ‘These marauders, these three, five, vagrant youths, are they going to kill me?’ (Interview #1)

The killings of Edip Solmaz and Ahmet Özdemir led to a decades-long violent feud between the PKK and the Raman.^11^ Yet, in retrospect, some doubt has been cast about the involvement of the Raman in the killing of Edip Solmaz:

Maybe they were involved, maybe not. Yet, after the killing, they [the PKK and the Raman] went after each other. It drew [them] into an armed conflict in which many were killed. Yet, some believe this conflict was the result of a lack of dialogue. A feudal sense of pride didn’t allow the Raman to say, ‘We had nothing to do with this assault’. […] It was pride. Why should an *ağa* of the Raman explain himself, how could he be held accountable by a bunch of lower-class kids? (Interview #9)

The remaining questions about who exactly pulled the trigger further highlight the opaque nature of state violence and bring attention to the ways in which the right to democratic claim making is constrained through various forms of state violence.

The killing of Solmaz and the 1980 military coup closed the possibilities of alternative ways of configuring citizenship. The PKK became even more definitively excluded from political channels. Following the mass arrests of its members, the PKK withdrew its remaining activists from Turkey to Syria, Lebanon, and then northern Iraq. One of the interviewees, at the time a high school student in Batman, recalled how the activists withdrew from Batman to the villages in the countryside and continued on towards the safety of the mountains.

‘The following days [after the 1980 coup] PKK sympathisers passed by our village. Some asked for weapons, others asked for food or a horse. They all moved into the mountains’ (Interview #18).

These were the political activists who had been the driving force behind the municipal election campaigns, escaping the country to subsequently come back as guerrillas. In exile, the PKK reorganised itself, taking the form of an insurgent movement, launching its armed struggle in 1984, under the command of the aforementioned Mahsum Korkmaz, the PKK cadre who had offered to escort Solmaz home on the night of his murder. This specific case in Batman is an illustrative and detailed example of the broader trend of violent escalation following the elimination of possibilities of non-violent political change (Goodwin 2001, 168–169; O’Connor 2021, 102; Romano 2006, 52).

The militants who left, later returned, but armed this time. On 15 August 1984, guerilla units of the PKK planned a triple attack on the towns of Eruh (Siirt province), Şemdinli (Hakkari province) and Çatak (Van province), though the latter was cancelled for operational reasons. Nevertheless, this was a large-scale, daring and well-coordinated attack that marked the beginning of a decades-long armed conflict (Çelik 2000, 71). Yet, although legal avenues have been repeatedly denied them by the authorities, the history of electoral and representational politics was not abandoned and continues to inspire the movement (Eren 2019; Özsökmenler 2014; Yaş 2019). In the 1990s, this legacy took a new form with the founding of the People’s Labour Party (Halkın Emek Partisi, HEP) by 15 Kurdish Members of Parliament (MPs). Expelled from the Socialdemocratic People’s Party (Sosyaldemokrat Halk Partisi, SHP) for attending a Kurdish conference in Paris, they responded by establishing a new political party. The Constitutional Court’s ban of HEP in 1993 marked the beginning of a recurring cycle of party closures and re-formations, reflecting the perception that pro-Kurdish politics threaten the unity of the state and its people (Koğacıoğlu 2004; O’Connor 2018). Over the past five decades, the Kurdish movement has persistently worked to expand political space and to frame the Kurdish issue as a political matter. In contrast, the state has continuously redefined it as a military and security concern, thereby repeatedly derailing efforts to establish a process for a peaceful resolution.

## Conclusion

We started this article by bringing to mind the call to focus on the *politics* of political violence (Staniland 2023), and the different questions and answers such a focus could bring forth (Worrall and Waterman 2023) about the foundational political dynamics of social movements (O’Connor and Oikonomakis 2015, 380). We argued that in particular the early stages of movement emergence, when political pathways are still inconclusive, have profound and lasting consequences. In this article, we looked at the politics of the PKK’s formative years. By analysing the PKK’s activism in the 1970s and its engagement in municipal politics, this article has provided two significant contributions and hinted at avenues for a broader research agenda related to Kurdish electoral mobilisation.

Firstly, although the literature on the nature of the Turkish state reveals much about the institutional limitations for democratic politics in the country, there is another, Kurdish-specific violent layer of oppression which has conditioned and limited all forms of pro-Kurdish mobilisation. This is an explicitly ethnically differentiated citizenship regime, in which the struggle for rights as Kurds is excluded from the political domain and considered a security threat. By focusing on one specific election campaign in Batman in 1979, the singular case serves as a revelatory window about how the state responded to concerted efforts by Kurds to establish themselves as political actors. This article shows that the micro-political and experiential aspects of political struggles in people’s lives deserve more attention. This implies a need to turn to local case studies, which include the hopes and expectations, the experiences and beliefs of ‘ordinary people’ and the activists in their struggles to establish themselves as holders of rights, as citizens.

Secondly, it calls into play the daily practices or everyday politics that question, challenge and change a citizenship regime that excludes people on the basis of their collective identity. By taking a local case, Batman, we were able to observe how ‘modernisation’ – industrialisation and migration, the emergence of an oil industry and the creation of an urban working class – strengthened traditional tribal dominance, but also fostered new forms of intra-elite rivalry. The cracks in the local elites–national parties framework created opportunities for the PKK to establish itself as a municipal political actor. The empirical basis of this article reveals much about the PKK as a movement, questioning linear understandings of its emergence as inevitably destined to become an insurgent movement. The article illustrates that representative and electoral politics preceded – by at least seven years – the PKK’s shift to insurgency. It was only in 1984, four years after the 1980 coup and the party’s reorganisation in Lebanon, that it turned to guerilla insurgency. The PKK had engaged in a much broader array of mobilisation, including the development of a counter-hegemonic model of Kurdish politics from within the limits of the state’s legal parameters. The data presented above shows not only *that* but also *how* the PKK claimed the right to stand for office as representatives of ordinary Kurds, through concerted attempts in the second half of the 1970s to participate in municipal elections. It was through these efforts the PKK challenged a restrictive citizenship regime and brought the voices, not only of Kurds but also of peasants, villagers, workers, and notably women, to municipal politics. We thus conclude that through the maintenance of a restrictive citizenship regime, in which Kurds can only become politically integrated as prospective Turks, the state denies any Kurdish potential to enact political change from within the system, resulting in the de facto separation of Kurdish politics from national politics, with all the attendant consequences witnessed over the last half century.

The long-term consequences of the state’s violent shutting of any avenues of political contestation short of armed insurgency have since scarred the region. And although the bloody decades of the 1980s and 1990s have limited collective memory of these specific forms of PKK mobilisation, the continuities with its reconfigured ideological model of Democratic Confederalism since the mid-2000s hint at alternative outcomes that could potentially have avoided the bloodshed (Jongerden and O’Connor 2023). The article also suggests the need for an expanded grounding of analysis of the Kurdish parliamentary tradition, predating the emergence of Kurdish parliamentary parties in the 1990s. The efforts outlined in this article attempted to redefine citizenship away from its Turkish ethnic definition and connected the struggle for Kurdish rights with broader citizenship campaigns centred on peasants, workers and women. This is reflected in the current politics of the Peoples Democracy and Equality Party (*Halkların Eşitlik ve Demokrasi Partisi*, DEM) and its call for inclusive citizenship, not only for Kurds, but also workers, peasants, LGTBQI+ and others. The 2023 metaphor used by imprisoned PKK leader Abdullah Öcalan to describe this party is telling: ‘Imagine a tree; can it have only one branch? A tree has many branches. Diversity is a universal rule’.^12^ This tree started to grow in Batman in 1979, but the state keeps on pruning its branches.

## Supplementary Material

Appendix A1.pdf

## Data Availability

The participants of this study did not give written consent for their data to be shared publicly, so due to the sensitive nature of the research supporting data is not available.

## Notes

1 The first refinery was built in 1944 with an initial daily capacity of 50 (later 200) tons; the construction of a new refinery began in 1951 and was completed in 1955 (capacity 1000 tons daily, 330,000 yearly, increasing to 580,000 by 1960 (Haytoğlu 2009).

2 After the 1960 military coup against President Adnan Menderes, a rift developed between various army factions, one of which opposed the junta’s plan to return to civil rule. Colonel Talat Aydemir, head of the Military Academy (*Kara Harp Okulu*) in Ankara, attempted coups in 1962 and in 1963, which both failed. As a student, Bağdu was under his command and participated in these coup efforts. Aydemir was hanged in 1964 (Esen 2021).

3 Although a party was established in November 1978, the organisation was renamed in the following April and its existence was only publicly announced in July 1979 through an assault on a ‘comprador’ landlord.

4 The network in Batman comprised figures such as Selahattin Celik, Idris Güzel, Mahsum Korkmaz, Ahmet Kurt, Mehmet Sener, and Mahmut Tanrıkulu (Interviews #1, #2, #3, #4). Other sources also mention Gurbet Aydın, Ahmet Ergin, Veysi Güzel, Ahmet İbin Mehmet, and Şah Deniz, most of whom were high school students at the time.

5 Mazlum Doğan was a member of the Central Committe of the PKK and editor of its journal, *Serxwebûn*, before becoming a important symbolic martyr within the PKK. He was born in 1955 in Teman, a village in the Karakoçan district of Elazığ province. Doğan studied to be a teacher at Eskişehir and Balıkesir before starting a course in economics at Hacettepe University in Ankara in 1974. He was arrested in 1979 and imprisoned in Diyarbakir Prison No. 5. He committed suicide by hanging on the evening of the Kurdish New Year, 21 March 1982. In PKK historiography, it is said that before killing himself, he lit three matches (symbolising the fire of Newroz, a major ritual celebration for Kurds, public observance of which was banned). His suicide is celebrated as an act of resistance against the torture he and other detainees were subjected to in prison. His death was interpreted as a symbol not to surrender to the daily tyranny or conform to the humiliating prison regime (PKK prisoners refused to wear prison uniforms, sing the national hymn or repeat the oath that one is ‘proud to be a Turk’).

6 In 1978, PKK member Selahattin Çelik had become a joint candidate of leftist and Kurdish parties for the presidency of the Petrol-Workers’ Union (*Petrol-İş Sendikası*) (Interviews #1, #17). His opponent was the far-right state-backed candidate Hüseyin Velioğlu, later leader of Kurdish Hizbullah, which operated as a state-affiliated anti-PKK death squad in the 1990s. This suggests that there have been elements of continuity between the state’s attempts to suppress the PKK politically in Batman in the 1970s and the 1990s’ counter-insurgency with its attendant mass human rights abuses (see Kurt 2017; O’Connor 2021). However, the Petrol-Workers’ Union congress for the election was cancelled after the imposition of martial law in several Kurdish regions in late 1978 (Interview #17).

7 Importantly, young radical groups like the emergent PKK were also disillusioned by the incrementalist strategy advocated by groups like the Workers Party of Turkey (*Türkiye İşçi Partisi*, TİP), which enjoyed substantial popular sympathy, especially amongst Kurds, but failed to substantially advance its agenda (Bozarslan 2012, 3). Although it rigorously played by the ‘rules of the political game’, it was crushed by the state with the same vehemence as more radical and violent organisations. Accordingly, the PKK advocated for a strategy of mass political mobilisation which crossed the bounds of municipal and extra-institutional militancy: marking a shift from the forlorn conspiratorial outlook of those awaiting salvation from allies in the military and the reformist perspective of electoral parties.

8 Baki Karer left the PKK in 1984 and became a fierce critic of the party (Jongerden and Akkaya 2011).

9 District village: the administrative centre of a rural district.

10 Korkmaz subsequently became a renowned PKK militant, and the PKK’s political and military training academy established in Lebanon was named after him.

11 The feud translates into ongoing political affiliations. Serkan Ramanlı is, today, vice president and a Member of Parliament of the Free Cause Party (*Hür Dava Partisi*, *Hüda*). Hüda is the political successor of the Kurdish Hizbollah, whose founder Hüseyin Velioğlu had competed with Selahattin Çelik for the position of president of the union for workers in the petrol industry in Batman. The party supported incumbent president Recep Tayyip Erdoğan in the 2018 and 2023 presidential elections.

12 http://mezopotamyaajansi35.com/tum-haberler/content/view/211090 (accessed 19 June 2023).
