# Supplementary material for: Contesting an exclusive citizenship regime: the Kurdistan Workers Party (PKK) and its electoral mobilisation in Batman in the late 1970s
Source: Third World Q. 2025 Jul 1;46(8):913–31. doi: 10.1080/01436597.2025.2518501 (PMC12312805; doi:10.1080/01436597.2025.2518501)
Supplement: Appendix A1.pdf [file CTWQ_A_2518501_SM9568.pdf]

Appendix A1
List of interviews

No. Interview	Gender	Description	Date(s) of Interview	Place of Interview
Interview 1	M	Former activist in Batman in the late 1970s	26-02-22 & 27-02-22	Netherlands
Interview 2	M	Former activist in Batman in the late 1970s	27-04-2022	Belgium
Interview 3	M	Former activist in Batman and Hilvan in the late 1970s	06-06-2022	Netherlands
Interview 4	M	An acquaintance of Edip Solmaz in the late 1970s	25-07-2022	Turkey
Interview 5	F	Witness of politics in Batman in the late 1970s	09-08-2022	Turkey
Interview 6	M	Active in politics in Batman in the late 1970s	09-08-2022	Turkey
Interview 7	M	Active in politics in Batman in the late 1970s	13-08-2022	Turkey
Interview 8	M	Expert in Turkish military affairs	19-08-2022	Online
Interview 9	M	Acquaintance of Edip Solmaz in the late 1970s	26-08-2022	Turkey
Interview 10	M	Expert in economic affairs,	30-08-2022	Online
Interview 11	M	Expert in economic affairs	31-08-2022	Online
Interview 12	M	Former activist in Batman in the late 1970s	22-09-2022	Turkey
Interview 13	M	Former activist in Batman in the late 1970s	06-10-2022	Turkey
Interview 14	M	Former activist in Hilvan in the late 1970s	08-11-2022	Belgium
Interview 15	M	Active in politics in Turkey in the late 1970s	13-08-2022	Turkey
Interview 16	F	Witness of politics in Batman in the late 1970s	13-12-2022	Netherlands
Interview 17	M	former activist in Batman in the late 1970s	13-12-2022	Belgium
Interview 18	M	High school student in Batman in the late 1970s	20-12-2022	Netherlands
Interview 19	M	Former activist in Hilvan in the late 1970s	02-01-2023	Online
Interview 20	M	Former activist in Batman and Urfa in the late 1970s	11-05-2023	Germany
Interview 21	M	High school student in Batman in the late 1970s	08-08-2023	Netherlands
Interview 22	F	Former activist in Hilvan in the late 1970s	11-08-2023	Turkey
Interview 23	F	former high school student in Hilvan in the late 1970s	27-08-2023 & 05-11-2023	Online
